# A Cross-Sectoral Telemedicine Network (sekTOR-HF) for Patients with Heart Failure

**DOI:** 10.3390/jcm14061840

**Published:** 2025-03-09

**Authors:** Sebastian Barth, Martina Hautmann, Wilko Reents, Goran Trajkovski, Brigitte Gebhard, Sebastian Kerber, Michael Zacher, Dimitar Divchev, Bernhard Schieffer

**Affiliations:** 1 Department of Cardiology, Cardiovascular Center Bad Neustadt, Von-Guttenberg-Straße 11, 97616 Bad Neustadt, Germany; 2 Department of Cardiac Surgery, Cardiovascular Center Bad Neustadt, 97616 Bad Neustadt, Germany; wilko.reents@campus-nes.de; 3 Medical Care Center Bad Neustadt GmbH, Von-Guttenberg-Straße 16, 97616 Bad Neustadt, Germany; 4 Department of Medical Documentation, Cardiovascular Center Bad Neustadt, 97616 Bad Neustadt, Germany; 5 Department of Cardiology, University of Marburg, 35043 Marburg, Germany

**Keywords:** cross-sectoral telemedicine network, sekTOR-HF, heart failure

## Abstract

**Objectives:** Heart failure is associated with frequent hospital admissions and high mortality. Digital medical technologies could help to improve information exchange between healthcare providers and patients to prevent recurrent cardiac decompensation. **Methods:** Eligible patients aged between 18 and 85 (mean age 65 ± 12; 35.4% female) with symptomatic heart failure were included in this cross-sectoral telemedicine network (sekTOR-HF) study (n = 79) with a 12-month intervention period. Depending on the severity of heart failure at the time of inclusion, patients in the intervention group were labeled either as inpatients (NYHA III–IV) or outpatients (NYHA I–II). All patients not included served as the control group. Nearest Neighbor Propensity Score Matching was performed to obtain a control group of the same size. Patients in the intervention group received an electronic patient record with all relevant health data in an eHealth portal and the option to use learning modules. A coordinating network office supported all patients in the intervention group. Monitoring included patient self-measurement of blood pressure, weight, heart rate, and oxygen saturation and a digital electrocardiogram. The primary endpoint was all-cause rehospitalization in both groups. **Results:** The cumulative incidence for all-cause rehospitalization was lower in the intervention group compared to the control group (sHR 1.86; 95% CI: 1.12–3.09). There was no difference in all-cause mortality (HR 1.5; 95% CI: 0.53–4.21). **Conclusions:** Intervention management in this cross-sectoral telemedicine network led to a lower cumulative incidence of all-cause rehospitalization even in the early phase of intervention.

## 1. Introduction

Heart failure (HF) is one of the most prevalent hospital admission causes associated with rising healthcare expenditures [1] and high mortality [2]. It is a condition that primarily affects older patients. In line with demographic changes, the number of hospital admissions due to HF has been steadily increasing in recent years [1]. Cross-sectoral and continuous care has been identified as a central requirement for the effective care of HF patients in actual national guidelines [3]. Numerous studies have shown that continuity of care is associated with fewer hospital admissions and reduced morbidity and mortality [4,5,6,7]. The lack of adequate communication and coordination strategies between healthcare providers, along with suboptimal discharge management, often prevents the assurance of care [3,8,9]. A pertinent structural problem also includes the absence of an electronic, patient-specific documentation system that provides information about previous diagnostic and therapeutic interventions. The resulting mismanagement is particularly reflected by the high number of ambulatory-sensitive hospital cases, meaning that there are avoidable hospitalizations of patients with mild heart failure (NYHA I–II) in Germany [10]. Conversely, the outpatient treatment of moderate to severe heart failure (NYHA III–IV) after hospital discharge often leads to rehospitalization within a month [11].

This research project, sekTOR-HF, was a prospective non-randomized cohort study of patients with symptomatic heart failure (HF) with a control group. The aim of our study was to investigate the association of temporary care for heart failure patients in the sekTOR-HF study over 12 months. The project was designed to overcome the current separation of inpatient and outpatient care in the healthcare system with the help of a telemedicine network and was funded by the Federal Government’s Innovation Promotion program (01NVF190006).

## 2. Methods

### 2.1. Study Population and Design of sekTOR-HF

**Figure 1:**
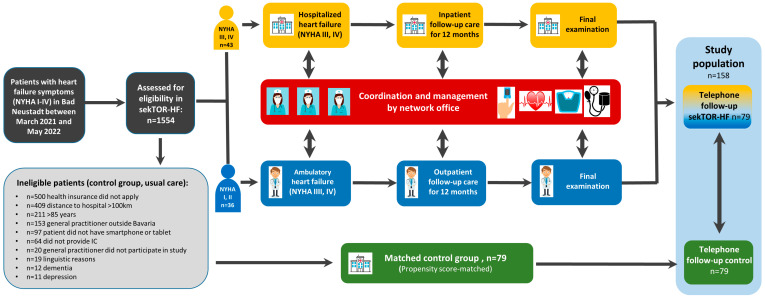
Flow chart of sekTOR-HF research project and control group.

Inclusion criteria for this sekTOR-HF research project were (1) heart failure symptoms (NYHA I-IV) and/or an elevated NT-proBNP in patients with NYHA class I (male < 50 years: <88 pg/mL; male >50 years: <277 pg/mL; female < 50 years: <153 pg/mL; female >50 years: <334 pg/mL), (2) an age between 18 and 85 years, and (3) residence within a radius of <100 km from the study hospital in Bad Neustadt. In addition, (4) only patients with a contract of a participating health insurance provider (Allgemeine Ortskrankenkasse AOK, Deutsche Angestellten-Krankenkasse DAK, or Techniker Krankenkasse TKK) were allowed to participate. Exclusion criteria included (1) dementia, (2) acute depression, (3) pregnant or breastfeeding women, (4) an age under 18 or over 85 years, (5) private health insurance, and (6) a lack of proficiency in German. All patients not included served as the control group. Nearest Neighbor Propensity Score Matching was performed to obtain a control group of the same size. The matching procedure is described in detail below. Depending on the severity of heart failure (HF) at the time of inclusion, patients in the intervention group were labeled either as inpatients (NYHA III–IV) or outpatients (NYHA I–II) (Figure 1). Treatment was performed according to the current ESC guidelines [2]. The division was maintained for the entire duration of the intervention (12 months). After the inpatient treatment ended, patients with initial NYHA III and IV remained in the inpatient group but could continue treatment on an outpatient basis. All participants received an electronic patient record with all relevant personal and health data in an eHealth portal. All patients in the intervention group had the opportunity to use learning modules on the eHealth platform with information about their condition, including behavioral instructions. These included further information on self-management such as a low-salt diet, daily weight monitoring, limiting fluid intake, behavior when traveling, medication adherence, and a physical exercise program. The use of these modules was optional and they were used by all patients. A coordinating network office (NWO) supported both patient groups (outpatient and inpatient care) throughout the entire project (Figure 1). A clinical specialist and HF nurses provided care via telemedicine or in the patients’ home environment, supported by digital tools. The NWO operated on weekdays between 7:30 and 16:00. During this time, phone inquiries were processed immediately. Written inquiries were answered within 2–3 h (the next day at the latest). Monitoring required the collection, structuring, and analysis of vital parameters (blood pressure, weight, heart rate, and oxygen saturation) and treatment-relevant data (ECG, medical documents) in the eHealth portal. The vital parameters were entered daily for the inpatient-treated patients (NYHA III–IV) and at least three times a week for the outpatient-treated patients (NYHA I–II). An ECG was digitally recorded at least once a week in both groups. In cases of issues with the electronic entry, the NWO made contact by phone and, if necessary, also conducted a personal visit. In coordination with the involved physicians, the NWO ensured that patients received the correct therapy. In the event of impending cardiac decompensation, the NWO coordinated individual measures with the involved outpatient/inpatient physicians. Two medical departments of the University Hospital Marburg and the Cardiovascular Center Bad Neustadt as the inpatient clinics, along with 18 outpatient medical care centers, were involved. The following analysis included only patients from Bad Neustadt. Information about the vital status and readmission rate or reason for readmission of each patient was obtained by phone from patients, relatives, medical records, or general practitioners after 12 months and then annually. After forming a control group, these patients, or their relatives or their general practitioner, were contacted by phone and asked for information about the survival status, as well as the time, frequency, and reason for any possible readmission. Hospitalization was defined as unplanned hospital admission with at least 1 overnight stay. This sekTOR-HF study was approved by the Ethics Committee of the State Medical Association of Hesse (2020-2179-evBO). All methods were carried out in accordance with the relevant guidelines and regulations of the Declaration of Helsinki.

### 2.2. Study Endpoints

The primary endpoint of this study was the all-cause rehospitalization rate in both groups. The analysis was performed on a time-to-first-event basis. The secondary endpoint was the all-cause mortality rate in both groups.

### 2.3. Statistical Analysis

Descriptive statistics for categorical variables were presented as absolute and relative frequencies, and those for continuous normally distributed variables were presented as teh mean and standard deviation (SD). Survival was analyzed using the Kaplan–Meier method, while differences between cohorts were tested for significance by COX regression. All data analyses were performed using IBM SPSS Statistics for Windows 2023, Version 29.0.2.0 (Armonk, NY, USA: IBM Corp. and R Core Team (2024); R: A Language and Environment for Statistical Computing; R Foundation for Statistical Computing, Vienna, Austria).

### 2.4. Propensity Score Matching

To form a control group of the same size, we conducted Nearest Neighbor Propensity Score Matching using the excluded patients. Since all patients in this sekTOR-HF research project were insured with the AOK, DAK, and TKK health insurance providers, the dataset in the control group was limited to these insurance providers. In the next step, parameters such as age, gender, body mass index, discharge type, place of residence/postcode, and marital status were derived. Based on the calculated score, each patient in the intervention group was matched with the nearest patient in the control group using the greedy matching method. The tolerable deviation of the propensity score for each match was defined without using a caliper. Therefore, all 79 cases from the intervention group were included. The matching effect was characterized using z-differences [12].

### 2.5. Cumulative Incidence

The Gray Test and the Fine–Gray model were used to compare the cumulative incidence function for readmission in our competing risk data models [13].

## 3. Results

### 3.1. Baseline Characteristics and Medical History

Between March 2021 and May 2022, 1554 patients who were referred to our clinic in Bad Neustadt due to heart failure symptoms were screened for participation in this sekTOR-HF study. Due to the predefined inclusion criteria (see above), the majority of patients could not be included. The reasons are detailed in Figure 1, with several of them potentially occurring simultaneously. The main reason for exclusion was insurance status with a health insurance provider that did not participate in this study (n = 500). Other reasons included excessive distance from the hospital (n = 409) and an age over 85 years (n = 211). Finally, 79 patients were included in the intervention group (n = 36 outpatient; n = 43 inpatient). All patients not included served as the control group (n = 1.475). Using Nearest Neighbor Propensity Score Matching, a statistical twin from the control group was assigned to each patient in the intervention group. Baseline demographic parameters, health insurance, marital status, and distance to the hospital were used as the basis for matching and were therefore well balanced. The patients in the intervention group had a higher proportion of dilated cardiomyopathy (DCM) (intervention: 55.7%; control: 31.6%), more mitral valve insufficiencies of grade ≥2 (intervention: 17.7%; control: 13.3%), more prior heart surgeries (intervention: 20.5%; control: 8.8%), and a higher EuroSCORE II (intervention: 9.9 ± 14; control: 5.5 ± 9). The patients in the control group had a higher proportion of tachycardiomyopathies (intervention: 30.4%; control: 50.6%) and severe aortic valve stenosis (intervention: 2.5%; control: 12.3%) (Table 1).

### 3.2. Medication

The proportion of patients taking angiotensin receptor neprisylin inhibitors (ARNIs) (intervention: 48.1%; control: 34.6%), sodium-dependent glucose co-transporter 2 (SGLT2 inhibitors) (intervention: 55.7%; control: 32.1%), and diuretics (intervention: 74.7%; control: 66.7%) was higher in the intervention group than in the control group. In contrast, patients in the control group were treated more frequently with angiotensin-converting enzyme/angiotensin receptor blocker (ACE/ARB) (intervention: 45.6%; control: 55.1%).

### 3.3. Compliance

Ninety-five percent of both outpatient (thirty-four out of thirty-six) and inpatient (forty-one out of forty-three) patients reliably entered their self-measured values without any reminders.

### 3.4. Follow-Up and Outcome

**Figure 2:**
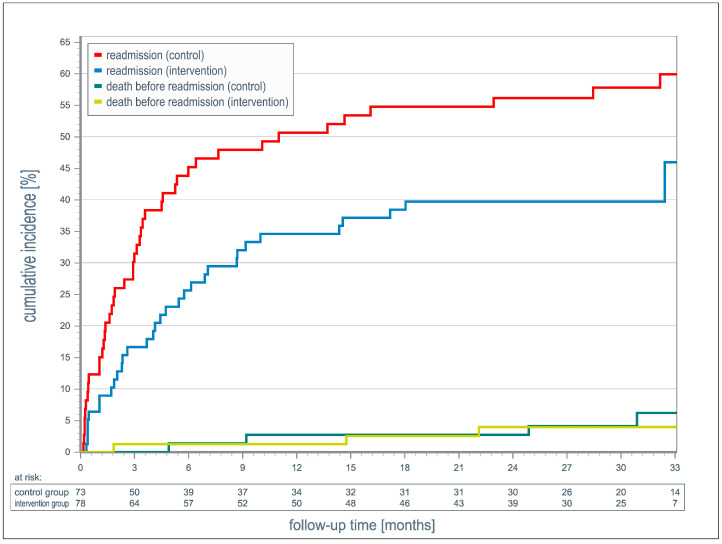
Association between 12 months’ temporary care with sekTOR-HF and usual care in all-cause hospitalization.

**Figure 3:**
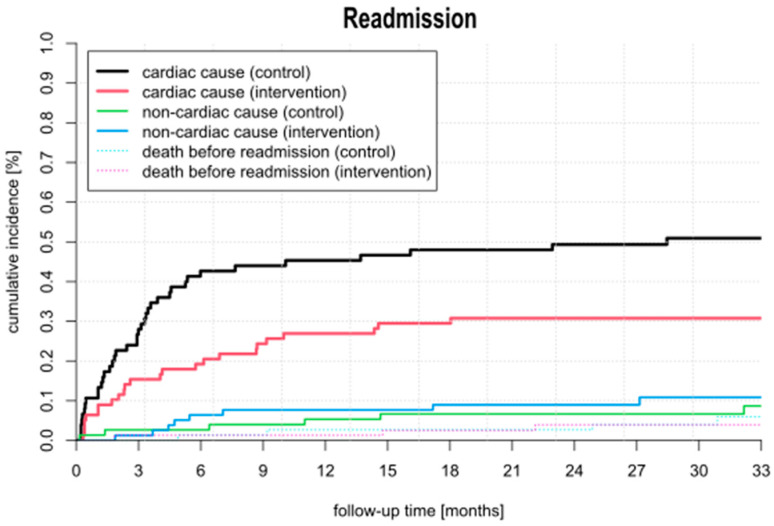
Association between 12 months’ temporary care with sekTOR-HF and usual care in rehospitalization divided by cardiac and non-cardiac cause.

The rehospitalization rate was significantly lower in the intervention group compared to the control group (sHR 1.86; 95% CI: 1.12–3.09). After 33 months, the cumulative incidence for rehospitalization was 59.9% in the control group (95% CI: 52.3–77.7%) and 46.0% in the intervention group (95% CI: 33.1–63.9%) (Figure 2). A significantly faster increase in the cumulative incidence of rehospitalizations was observed in the control group compared to the intervention group. The median time to first re-hospitalization was longer in the intervention group (intervention: 4.4 months; control: 3.1 months). The cumulative incidence of rehospitalization for cardiac reasons after 33 months was significantly higher in the control group than in the intervention group (control: 50.9%; intervention: 30.8%; *p* = 0.010) (Figure 3). The proportion of non-cardiac readmissions was comparable in both groups (control: 6.6%; intervention: 10.8%; *p* = 0.49) (Figure 3).

**Figure 4:**
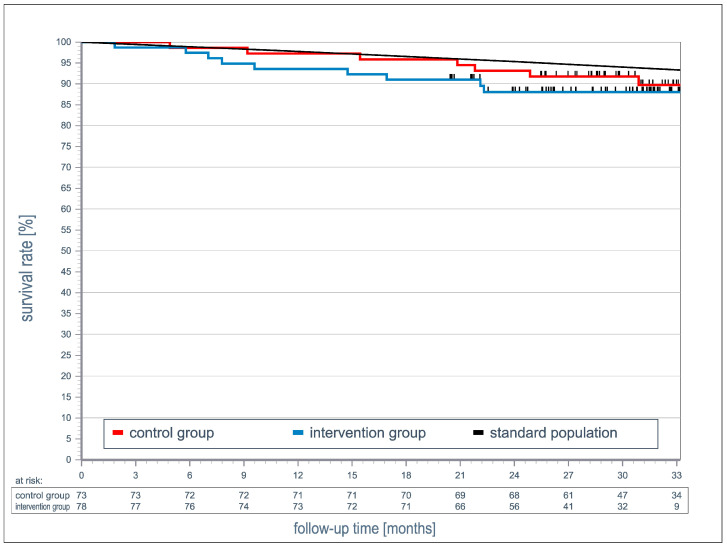
The association between 12 months’ temporary care with sekTOR-HF and usual care in all-cause death. The black line shows the survival rate of the general population in Germany according to the Federal Statistical Office [14].

A total of 18 patients died during follow-up, with 9 in each group (HR 1.5; 95% CI: 0.53–4.21). The deceased patients in the intervention group died on average 9 months earlier than those in the control group (Figure 4).

## 4. Discussion

In this study, the addition of a cross-sectoral telemedicine network was compared to standard care for patients with heart failure. The core element of this form of care was an eHealth platform that enabled significantly improved and faster information exchange between all involved healthcare providers. At the same time, participating patients had the opportunity to receive information about their condition through optional learning modules. The main findings were as follows: (1) Patients in the intervention group had a significantly lower cumulative incidence of all-cause rehospitalizations compared to those in the control group, with this association being detectable even in the early phase of the intervention. (2) Rehospitalization due to cardiac causes was significantly higher in the control group. (3) The time to the first hospital admission was longer in the intervention group than in the control group. (4) There were no differences between the two groups regarding mortality.

### 4.1. Rehospitalization

In the control group, there was a significantly faster increase in the cumulative incidence of rehospitalization compared to that in the intervention group. With the same proportion of non-cardiac reasons, rehospitalization due to cardiac causes was significantly higher in the control group. Furthermore, the time to the first hospital admission was shorter in the control group than in the intervention group. The frequency of death prior to readmission was very low and practically identical in both groups. It can be definitively ruled out that the reduction in hospital readmissions in the intervention group was achieved at the expense of increased deaths.

In the HeartNetCare-HF [15] study, no significant difference between the two groups regarding death or hospitalization was observed after the 18-month intervention and after the 60-month follow-up. This difference was detectable only after an extended follow-up period of up to 120 months. To achieve a long-lasting positive effect on health through a temporary multimodal intervention, high patient compliance with self-monitoring and the derived therapy is necessary. This requires a certain level of disease understanding and awareness [15]. The CONNECT-HF study [16] evaluated the effect of a hospital- and post-discharge-level intervention compared with usual care. Patients with a smartphone had the ability to use a mobile application to facilitate the improved use of guideline-directed recommendations for the self-monitoring and self-management of activity and medications. This offer was optional in the CONNECT study. The main concern was that the mandatory use of a digital app could have led to a significant limitation in the patients eligible for inclusion. The hospital and post-discharge quality-improvement intervention was led by a group of HF and quality leaders who underwent specialized training for the CONNECT study. There was no significant difference in time to first heart-failure rehospitalization or death between the two groups after a 12-month follow-up. Because the clinicians who enrolled patients were often not the ones providing patient follow-up after discharge, the hospital-based study team had limited ability to influence the care after discharge. In contrast to the HeartNetCare-HF [14] and E-INH [17] studies and partially to the CONNECT study [16], digital medical technologies in the form of an eHealth platform were available in sekTOR-HF and usage was mandatory. Participation in this seKTOR-HF research project required, in any case, a certain level of affinity for technology among these patients. Only patients with the capacity to use mobile applications on a smartphone were eligible for enrollment. This requirement was not present in all patients and often served as an exclusion criterion. For this form of care to be successful, the motivation of the patients to actively engage in self-monitoring and the therapy being carried out is therefore a crucial prerequisite. In the TEN-HMS study [18], four-fifths of the patients with telemonitoring made at least one daily measurement (weight or blood pressure), and approximately half had an 80% compliance with twice-daily measurements. In our study, the daily self-entry of data by inpatient patients and the three-times-weekly entry by outpatient patients were even higher and occurred in 95% of the cases, demonstrating high motivation. We therefore agree with the assessment of Angermann et al. that patient selection and motivation played an important role in the success of this tailored multimodal intervention [17].

Participating patients had the opportunity to use learning modules to gain information about their condition and treatment measures. In addition to the intensive care provided by the NWO, this likely contributed to an increase in patient motivation regarding the recommended and implemented therapy. It seems plausible that this improved health literacy had a positive impact on health maintenance and, through enhanced therapy compliance, supported the positive effects of this treatment approach.

In addition to improved disease understanding, the eHealth platform enabled an improved and faster exchange of information between all participating healthcare providers in both the inpatient and outpatient settings. For this reason, suitable measures could be initiated in an early stage in the event of impending cardiac decompensation. This may be the reason why, in our study, a reduction in the cumulative incidence of rehospitalizations and the extended time to the first readmission were detectable earlier compared to those in the E-INH [17] and CONNECT studies [16]. The TEN-HMS study demonstrated that the duration of HF-related admissions in high-risk outpatients could only be reduced if nurse telephone support was combined with the twice-daily electronic transmission of self-monitoring results to caregivers [18].

The use of ARNIs and SGLT2 inhibitors was not clinically routine at the time of the HeartNetCare-HF [14] and E-INH [17] studies. These drugs represent an important advancement in the treatment of heart failure patients and have become an integral part of clinical practice [2]. The proportion of ARNIs and SGLT2 was higher in our intervention group than in the control group. The low use of guideline-directed medical therapy is a common issue in the treatment of patients with HFrEF in clinical practice and in studies [19]. The Change the Management of Patients With Heart Failure (CHAMP-HF) registry [20] showed that ACE inhibitors, ARBs, and ARNIs were titrated to the target dose in only one-quarter to one-fifth of patients, and less than 1% of patients received all three medications. Therefore, one possible explanation for the lower rehospitalization rate in our intervention group could also be related to the better cardiovascular heart failure medication. In contrast to the TIM-HF2 study [21], where medication up-titration and measures to strengthen self-care were not part of the intervention, in our study, the pharmacological heart failure therapy was closely adjusted via the NWO. The fact that patients in our intervention group were sicker overall, with a higher proportion of DCM, more mitral valve insufficiencies as a potential cause of cardiac decompensations, more prior heart surgeries, and a higher EuroSCORE II, highlights the positive association of this type of care.

The study by Mariani et al. demonstrated that virtual visits for cardiac electrophysiology patients did not lead to an increase in remote monitoring alerts or emergency hospital admissions. At the same time, high patient satisfaction was documented [22]. Particularly in light of demographic change and the increasingly limited resources in healthcare, a hybrid model of care including virtual visits represents a promising form of care for the future [22].

### 4.2. All-Cause Mortality

The telemedical intervention management in the intervention group did not lead to a reduction in mortality during the 33-month follow-up period compared to in the control group. Our 12-month intervention period was comparable to that in the TIM-HF2 study and 6 months shorter than that in the E-INH study [3]. In the TIM-HF2 study, the positive effects on mortality and morbidity could no longer be demonstrated after a 12-month extended follow-up after stopping the intervention [23]. Similarly, in the TEMA-HF study [24], no mortality difference was observed after 6.5 years following a 6-month telephone monitoring period. In contrast, both the E-INH [17] and TEN-HMS [18] studies demonstrated a mortality benefit in the intervention group after an extended follow-up period of 120 months (E-INH) and 1 year (TEN-HMS), respectively. Although no overall difference in mortality was observed in our study, the deceased patients in the intervention group died on average nine months earlier than those in the control group. This fact could be related to the possibility that the participants in the treatment group were sicker at the time of study inclusion than those in the comparison group.

### 4.3. Study Limitations

Due to the sekTOR-HF study protocol, the treatment cohort included patients who were either hospitalized or treated on an outpatient basis. The control group consisted solely of inpatient cases. This significant structural difference represents a limitation of this work. Due to the strict inclusion criteria, which required patients to be insured with one of the three participating health insurance companies, only a limited number of patients could be included.

Since only patients who owned a smartphone and were able to use mobile applications were included in this study, the effects of this intervention were limited to a highly select group of patients and cannot be generalized.

In the analysis of survival status, there were no data available for six cases in the control group and one case in the intervention group. Therefore, two sensitivity analyses were conducted. In the first step, it was assumed that all unknown entries corresponded to survivors. The mean follow-up time of the survivors was then used for the follow-up period. In the second step, the opposite assumption was made, and all unknown status entries were filled with “deceased”. For the time variable, the mean of the definitely deceased participants was used in these cases. Overall, no detectable mortality differences were found between the groups, either with the original data or the two described extreme scenarios (all unknowns were alive versus all unknowns were deceased). As a result, it can be assumed that inclusion in sekTOR-HF did not affect mortality in the affected participants during the first 33 months.

Death represents a competing event to rehospitalization. The frequency of death before readmission to the hospital was very low and practically identical in both groups. In analogy to the analysis of survival status, two sensitivity analyses were conducted for the seven missing cases. In the first analysis, it was assumed that these individuals were *alive*, and in the second, *death* was assumed for all unknown status entries. The estimate for the cumulative incidence of rehospitalization remained stable in both sensitivity analyses.

## 5. Conclusions

Patients in the intervention group had a significantly lower cumulative incidence of all-cause rehospitalization compared to usual care in our clinic. Rehospitalization due to cardiac causes was significantly higher in the control group. The time to the first hospital admission was longer in the intervention group than in the control group. There were no differences between the two groups regarding mortality. The prerequisites for these positive associations were (1) the possibility of rapid information exchange on the eHealth platform between all participating healthcare providers, (2) the prompt initiation of the appropriate therapy in an early stage of cardiac decompensation, (3) the improved health literacy of the patients, (4) the close monitoring and optimization of heart failure medications by the healthcare providers, and (5) a certain level of affinity for technology among these patients.

Due to changing demographics and increasingly limited healthcare resources, tailored telemedicine care presents a promising option for treating patients with physical frailty and significant geographical distance from medical providers in medically underserved areas, especially in rural regions.

## Figures and Tables

**Table 1:** Patient Demographic and Clinical Characteristics at Baseline.

	Total (n = 158)	Control (n = 79)	Intervention (n = 79)	Squared Z-Differences
**Demographic**				
Age, y		65 ± 13	65 ± 12	0.0193
Female	56 (35.4)	28 (35.4)	28 (35.4)	0.0000
Health Insurance				
AOK	98 (62.0)	49 (62.0)	49 (62.0)	0.0000
DAK	26 (16.5)	13 (16.5)	13 (16.5)	0.0000
TKK	34 (21.5)	17 (21.5)	17 (21.5)	0.0000
Marital Status				
single	18 (11.4)	9 (11.4)	9 (11.4)	0.0000
married	113 (71.5)	57 (72.2)	56 (70.9)	−0.1763
widowed	25 (15.8)	13 (16.5)	12 (15.2)	
divorced	2 (1.3)	0 (0.0)	2 (2.5)	
Distance to the clinic (km)		47.1 ± 47.3	50.0 ± 37.3	0.4157
**Heart Failure Symptoms**				1.0146
NYHA functional Class I	5 (3.2)	2 (2.5)	3 (3.8)	
NYHA functional Class III	62 (39.2)	29 (36.7)	33 (41.8)	
NYHA functional Class III	80 (50.6)	41 (51.9)	39 (49.4)	
NYHA functional Class IV	11 (7.0)	7 (8.9)	4 (5.1)	
**Comorbidities and Medical History**				
Coronary artery disease	91 (57.6)	43 (54.4)	48 (60.8)	0.8066
PCI	54 (34.2)	26 (32.9)	28 (35.4)	
Cardiac Surgery				
CABG	11 (7.0)	2 (2.5)	9 (11.5)	
Heart Valve Surgery	7 (4.5)	2 (2.5)	5 (6.4)	
Combined Surgery (CABG + Valve)	4 (2.5)	3 (3.8)	1 (1.3)	
LVAD	1 (0.6)	0 (0.0)	1 (1.3)	
M-TEER	2 (1.3)	2 (2.5)	0 (0.0)	
Peripheral vascular disease	12 (7.6)	6 (7.6)	6 (7.6)	0.0000
Hypertension	154 (97.5)	76 (96.2)	78 (98.7)	1.0162
Atrial Fibrillation	65 (41.1)	34 (43.0)	31 (39.2)	−0.4854
Atrial Flutter	8 (5.1)	1 (1.3)	7 (9.1)	2.2298
Supraventricular Extrasystoles	2 (2.5)	0 (0.0)	2 (1.3)	1.4325
Ventricular Extrasystoles	27 (17.2)	6 (7.6)	21 (26.9)	3.3095
Pacemaker and/or ICD	54 (34.2)	26 (32.9)	28 (35.4)	0.3356
COPD	18 (11.4)	6 (7.6)	12 (15.2)	1.5132
Hypercholesterolemia	128 (81.0)	61 (77.2)	67 (84.8)	1.2228
Diabetes mellitus	45 (28.5)	22 (27.8)	23 (29.1)	−0.1763
Dietary therapy	0 (0.0)	0 (0.0)	0 (0.0)	
Oral Hypoglycemic Agents	21 (13.3)	13 (16.5)	12 (15.2)	
Insulin	25 (15.8)	14 (17.7)	11 (13.9)	0.4789
Depression	6 (3.8)	3 (3.8)	3 (3.8)	0.0000
Family history of premature CHD	22 (13.9)	12 (15.2)	10 (12.7)	
Smoker (last 2 month)	20 (12.7)	10 (12.7)	10 (12.7)	
Former smoker	24 (15.3)	13 (16.5)	11 (14.1)	
Alcohol Abuse	3 (1.9)	0 (0.0)	3 (3.8)	1.7659
Myocarditis	9 (5.7)	4 (5.1)	5 (6.3)	
Stroke	12 (7.6)	8 (10.1)	4 (5.1)	
ICM	81 (51.3)	41 (51.9)	40 (50.6)	
DCM	69 (43.7)	25 (31.6)	44 (55.7)	
HCM	2 (1.3)	2 (2.5)	0 (0.0)	
HOCM	0 (0.0)	0 (0.0)	0 (0.0)	
Toxic CM	4 (2.5)	1 (1.3)	3 (3.8)	
Tachycardiomyopthy	64 (40.5)	40 (50.6)	24 (30.4)	
**Measurements**				
EuroSCORE II	7.7 ± 10	5.5 ± 9	9.9 ± 14	
BMI, kg/m^2^	31.1 ± 10	30.6 ± 6	31.6 ± 15	0.5810
LVEF, %	39.5 ± 14	41 ± 15	38 ± 13	−1.2405
LVESD	46 ± 11	44 ± 13	46 ± 11	
LVEDD	55 ± 9	52 ± 9	58 ± 9	
sPAP	33 ± 12	31 ± 9	34 ± 13	
Mitral Valve Regurgitation, grade ≥2	24 (15.5)	10 (13.3)	14 (17.7)	
Aortic Valve Stenosis, grade 3	11 (7.2)	9 (12.3)	2 (2.5)	
Tricuspid Valve Regurgitation, grade ≥3	3 (1.9)	2 (2.7)	1 (1.3)	
**Laboratory values**				
Creatinine, mg/dl	1.14 ± 0.5	1.15 ± 0.5	1.13 ± 0.4	−0.2228
NT-proBNP, pg/ml	2222 ± 3196	2346 ± 2995	2098 ± 3397	−0.4037
Glomerular Filtration Rate, ml/min/1.73m^2^	70.6 ± 30	73.1 ± 41	68.0 ± 22	−0.9756
**Discharge Medication**				
ACE/ARB	79 (50.3)	43 (55.1)	36 (45.6)	
ARNI	65 (41.4)	27 (34.6)	38 (48.1)	
Beta-blocker	154 (98.1)	27 (97.4)	78 (98.7)	
Diuretic	111 (70.7)	52 (66.7)	59 (74.7)	
MRA	83 (52.9)	39 (50.0)	44 (55.7)	
SGLT2 Inhibitor	69 (43.9)	25 (32.1)	44 (55.7)	
Calcium Antagonist	25 (15.9)	19 (24.4)	6 (7.6)	
Statin	115 (98.1)	58 (74.4)	57 (72.2)	
Ivabradine	3 (1.9)	1 (1.3)	2 (2.5)	
Digitalis	2 (1.3)	1 (1.3)	1 (1.3)	
OAC	74 (46.8)	37 (46.8)	37 (46.8)	
Cumarine	23 (14.6)	8 (10.1)	15 (19.0)	
DOAC	51 (32.2)	29 (36.7)	22 (27.8)	

Values are n (%), mean ± SD. ACE: Angiotensin Converting Enzyme, ARB: Angiotensin Receptor Blocker, ARNI: Angiotensin Receptor Neprilysin Inhibitor, BMI: Body Mass Index, CABG: Coronary Artery Bypass Grafting, CHD: Coronary Heart Disease, COPD: chronic obstructive pulmonary disease, CM: Cardiomyopathy, DCM: Dilated Cardiomyopathy, DOAC: Direct Oral Anticoagulation, HCM: Hypertrophic Cardiomyopathy, HOCM: Hypertrophic Obstructive Cardiomyopathy, ICD: implanted cardioverter-defibrillator, ICM: Ischemic Cardiomyopathy, LVAD: Left Ventricular Assist Device, LVEDD: Left Ventricular Enddiastolic Diameter, LVESD: Left Ventricular Endsystolic Diameter, LVEF: Left Ventricular ejection fraction, M-TEER: Mitral Valve Transcatheter Edge To Edge Repair, MRA: Mineralcorticoid Receptor Antagonist, NYHA: New York Heart Association, OAC: Oral Anticoagulation, PCI: Percutaneous Coronary Intervention, SGLT2: Sodium-Dependent Glucose Co-Transporter 2, sPAP: Systolic Pulmonary Artery Pressure.

## Data Availability

Data are available from the corresponding author upon reasonable request.
